# Effect of adherence to growth hormone treatment on 0–2 year catch-up growth in children with growth hormone deficiency

**DOI:** 10.1371/journal.pone.0206009

**Published:** 2018-10-24

**Authors:** Paula van Dommelen, Ekaterina Koledova, Jan M. Wit

**Affiliations:** 1 Department of Child Health, TNO, Leiden, The Netherlands; 2 Global Medical, Safety & CMO, Merck KGaA, Darmstadt, Germany; 3 Department of Pediatrics, Leiden University Medical Center, Leiden, The Netherlands; McMaster University, CANADA

## Abstract

**Background:**

Quantifying the association between adherence and the growth response to growth hormone (GH) treatment is hampered by suboptimal methods of measuring adherence, confounders associated with the growth response, and restriction of the outcome parameters to yearly growth velocities.

**Aim:**

To investigate the effect of adherence on the two-year growth response to GH treatment in prepubertal children with idiopathic isolated growth hormone deficiency (GHD) participating in the easypod connect observational study (ECOS), a 5-year, Phase IV open-label study to continuously assess real-world adherence via the easypod electronic drug-delivery device.

**Patients and methods:**

Outcome measures were change in height standard deviation score (ΔHSDS), index of responsiveness (IoR), and parameters of two catch-up growth (CUG) curve functions (monomolecular growth curve and second degree polynomial) with adj-HSDS (HSDS minus Target height (TH) SDS) as dependent variable. Inclusion criteria were GHD, naïve to GH treatment, known TH, age <10y in girls and <12y in boys, ≥3 measurements, HSDS <-2 at start, complete data on growth and adherence in the first and second year. Linear regression analyses were performed to test the association between adherence (continuous and high vs. low) and the outcome measures, also adjusted for potential clinical confounders (age at start, adj-HSDS at start, birth weight SDS, gestational age (<37 weeks vs ≥37 weeks), GH dose, GH max (n = 58)). The formula of IoR already adjusts for confounders.

**Results:**

In total, 95 patients complied with the inclusion criteria. The strongest associations were found between high adherence in the second year (≥91% as cut-off value) and IoR 2y (+0.62), and average adherence and high adherence (≥78%) in the first two years and ΔHSDS 0-2y (+0.11 SD per 1 injection/week, and +0.34 SD for high vs. low adherence).

**Conclusion:**

Suboptimal adherence negatively affected the growth response in the first two years of GH treatment.

## Introduction

Growth hormone (GH) treatment for children with GH deficiency (GHD) is efficacious in generating catch-up growth (CUG) in the first years of treatment, usually followed by a maintenance phase and finally leading to a height close to the target height (TH) [1,2]. However, the growth response has been shown to be quite variable. Several groups have investigated the clinical features associated with the growth response [3,4,5,6], and in general approximately 50% of the variance could be explained by features like the severity of GHD (as assessed by the GH peak in provocation tests), age, bone age delay, birth weight, etc [7].

None of these prediction models could include adherence into their models, because there were no tools to assess adherence accurately. However, several studies have shown that adherence has an important effect on growth on GH treatment [8,9,10,11,12], although they could only be conducted over short time periods, and using a methodology based on self-reporting or number of issued and renewed prescriptions.

There are several challenges in studying the association between adherence to growth hormone (GH) treatment versus the growth response to GH. The first challenge is to make a decision on which growth parameter is the most suitable outcome parameter. So far, most studies investigating predictive factors have used yearly growth velocity data, particularly first year’s height velocity [3,6]. Others have used the change in height Standard Deviation Score (HSDS) (delta HSD, ΔHSDS) over 1 or 2 years [4,5]. All these parameters depend on the age and height SDS at start of treatment, and do not fully represent the actual shape of the catch-up growth (CUG) in the first years of GH treatment in children with GHD. The second challenge is that, besides adherence, there are many other clinical parameters that influence the growth response to GH, as illustrated by the various prediction models [7]. So, in order to obtain an unbiased effect of adherence, it is necessary to adjust for important clinical predictors that are confounders in an analysis of the effect of adherence on the growth response to GH. The third challenge is to find a reliable method for measuring adherence.

In this study we accounted for these challenges. First, we used a novel way of expressing CUG on GH treatment, using mathematical models of HSDS (adjusted for TH]) over two years of GH treatment. In an earlier study, we showed that mathematical modelling of CUG with the monomolecular growth curve is suitable for celiac disease [13]. A second degree polynomial was also used, because for most children the shape of the HSDS in the first two years is a catch-up in growth followed by stabilization. Second, we entered important potential confounders into the models. Third, we used a novel electronic tool to assess adherence accurately (easypod) [14,15,16]. For this purpose we used data from children naïve to GH treatment participating in the ECOS study, using automated continuous assessment of adherence through easypod. The easypod Connect observational study (ECOS) is a 5-year, Phase IV open label study that started in 2010 in 24 countries to assess ‘real-world’ adherence via the easypod electronic drug delivery device.[17]

## Methods

### Data

Adherence data were derived from the easypod device combined with physician data entry of outcome measures. Data were collected retrospectively and prospectively. Collected data were analyzed in a multinational pooled analysis.

Inclusion criteria for the ECOS study were: GH administered via the easypod electromechanical device; under 18 years of age, or over 18 without fusion of growth plates; parent’s or guardian’s written informed consent, given before entering data into the registry/observational study (if the child was old enough to read and write, a separate assent form was given as defined in the appropriate jurisdiction of each country). Exclusion criteria for the ECOS study were: Subjects taking GH in whom growth plates have fused (i.e. taking GH for its metabolic effects); contra-indications to Saizen GH; Use of an investigational drug or participation in another interventional clinical study. The study was conducted in accordance with the principles of the Declaration of Helsinki, Good Clinical Practice (ICH-GCP E6) guidelines and applicable national legal and regulatory requirements.

To investigate the effect of adherence on the two-year growth response to GH treatment in prepubertal children with idiopathic isolated growth hormone deficiency (GHD) in the ECOS study, the following selection criteria were applied:

- Naïve to GH treatment
- Patients diagnosed with Idiopathic Isolated GHD by their physicians
- Known parental heights, so that TH could be calculated
- Age at end of 2 year study period <10 years in girls and <12 years in boys (i.e. before onset of puberty)
- At least three measurements of height available from start treatment
- HSDS <-2.0 at start treatment
- At least two data measurements on growth and adherence in the first and second year after start of treatment

The age limit was used for two reasons. First, we wished to limit the analysis to prepubertal children, because insufficient data were available in the database about pubertal status during GH treatment. Second, beyond these age limits the SD of the population’s growth reference charts shows a non-linear pattern of an increase followed by a decrease, due to the varying ages at pubertal onset in the general population. Because HSDS is the main outcome parameter in our analysis, a higher cut-off for age would generate noise and bias.

### Statistical analyses

Adherence was calculated as the number of injections received divided by the number of planned injections during the considered period, expressed as a percentage. All adherence rate analyses were based on periods of complete weeks. Data are presented over the first two years.

Outcome measures for the growth response to GH treatment in the first two years were:

- Change in HSDS in the first (ΔHSDS 0–1 y), second (ΔHSDS 1–2 y) and first two years (ΔHSDS 0–2 y)
- Index of responsiveness (IoR) in the first and second year [3], where IoR first year = (height velocity first year-(12.41–0.36*Age at start GH+0.47* Birthweight SDS+1.54*(log(3*GH dose at start GH (mg/kg/wk)))-0.6*(HSDS 1y-TH SDS)+0.28*weight SDS 1y))/1.72, and IoR second year = (height velocity second year-(5.69–0.09*Age at start GH+0.63*(log(3*GH dose at start GH (mg/kg/wk)))+0.24*weight SDS 2y+0.31* height velocity first year))/1.19
- Parameters of the monomolecular growth curve for catch-up growth [13]: Monomolecular growth curve: adj-HSDS = A * (1—B * exp(-k * x))– 5, with x age after start GH
- Parameters of a second degree polynomial: second degree polynomial: adj-HSDS = c + D*x + E*x^2^, with x age after start GH

Mixed-effect models were used to fit the monomolecular growth curve and the second degree polynomial on adj-HSDS (HSDS minus TH-SDS). Each curve summarizes the first phase of CUG in three parameters. For the monomolecular growth curve, these parameters are A-5 = attained adj-HSDS after two years, A*(1-B)-5 = adj-HSDS at start, k = growth rate. For the second degree polynomial, these are c = intercept (adj-HSDS at start), D = slope (CUG), E = deceleration (deceleration part after CUG). A higher value of D implies a steeper curve between 0–2 years and a higher value of E implies a stronger CUG after approximately 1 year. If E >0, then there is a stronger CUG in the second year, and if E<0 then CUG is stronger in the first year.

Linear regression analyses were performed to test the association between adherence in the first, second, and the first two years and the outcome measures. Adherence is highly skewed with peak values near or at 100%. We therefore analyzed this independent variable both as a continuous variable and categorized into a high and low level. Recursive partitioning [18] in software R (package rpart) was used to find the cut-off point (the first split) for high and low adherence that maximizes the correlation between adherence and height gain.

The parameter B from the monomolecular growth curve and c from the second degree polynomial were not used in the analyses as outcome measures, because these are related to the adj-HSDS at start and only of interest as a potential confounder. We adjusted the associations for potential clinical confounders that were significantly related to adherence in the first, second or first two years. The potential clinical confounders were age at start, adj-HSDS at start (observed data, not derived from the monomolecular growth curve model), birth weight SDS, gestational age (<37 weeks vs. ≥37 weeks), GH dose, and GH max. The formula of IoR already adjusts for confounders. We, therefore, did not adjust the IoR for the potential clinical confounders. HSDS at exactly 1.0 and 2.0y were obtained by linear interpolation using the nearest measurements around these ages. WHO growth references were used to calculate the SDS values. These references were obtained from the WHO Multicentre Growth Reference Study (MGRS) (ages 0–5 years) and the WHO 2007 reference (5–19 years for height) [19]. TH-SDS was calculated as 0.72 x (father’s HSDS + mother’s HSDS)/2 [20]. This definition corrects for assortative mating and parent-offspring correlations. We also used the first and second year Index of Responsiveness (IoR) for the KIGS prediction model as outcome measures [3], but using the WHO references for weight SDS at birth.

## Results

**Fig 1:**
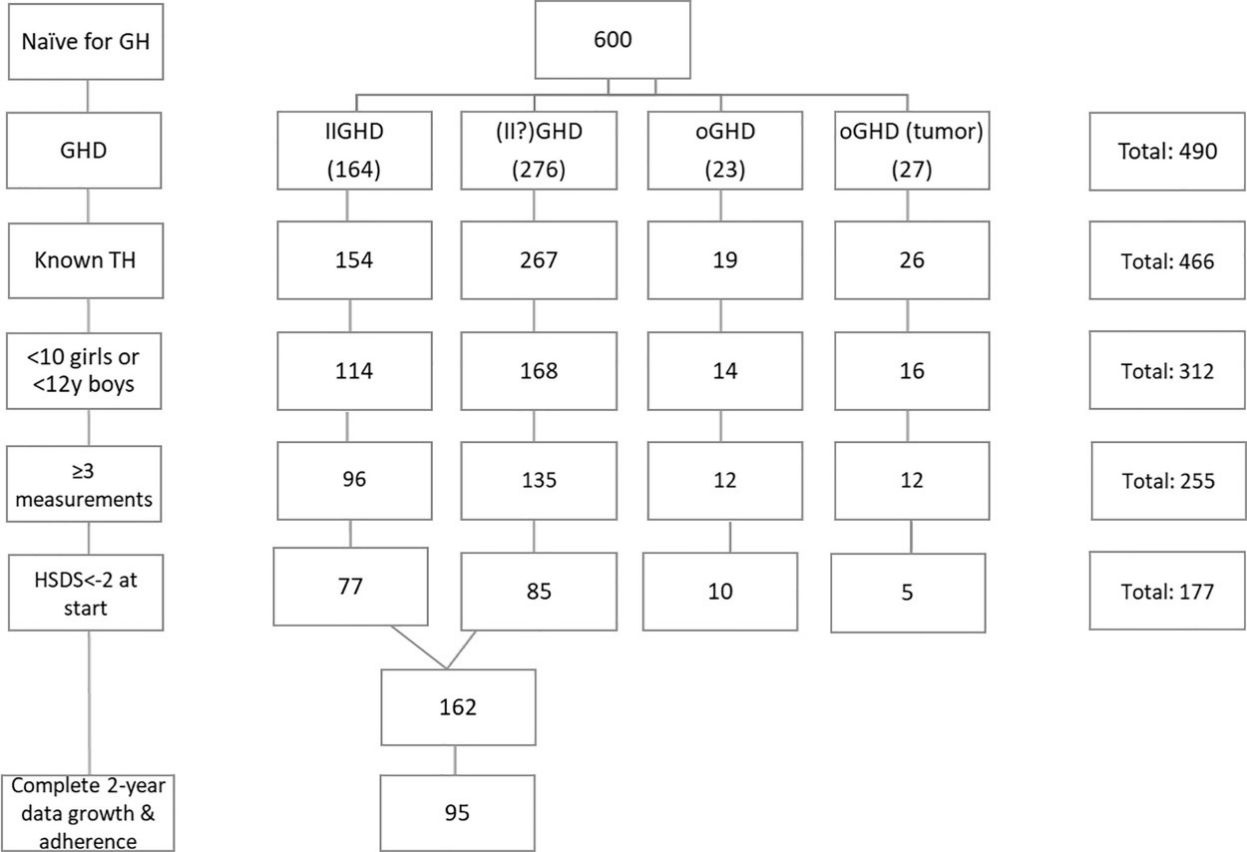
Selection criteria within the ECOS study.

Fig 1 shows the sample size of this study after applying the inclusion criteria. In total 490 naïve patients with GHD were available within the ECOS study. The sample size of patients with an Organic GH deficiency (OGHD) due to congenital/anatomical or tumor origin was low and therefore excluded from the analysis in order to analyse a homogeneous group. In total, 95 patients with Idiopathic Isolated GH deficiency (IIGHD) were available for analysis. Table 1 shows the descriptive statistics of the background characteristics and the clinical parameters of these 95 patients. Although one patient exceeded the (arbitrary) cut-off of 10 ug/L at the GH peak after stimulation (GH peak-1 = 9.97 and GH peak -2 = 12.5 ug/L), we included this patient in our study because the treating physician interpreted this patient as GHD.

**Table 1:** Background characteristics and clinical parameters of the sample (n = 95).

Characteristics	Mean (SD)	Median (min, max)	N (%)
Sex (male)			72 (76%)
Gestational age (<37 wks)			12 (13%)
Birth weight SDS	-1.0 (1.5)	-0.9 (-6.4, 1.2)	
Age at start	6.3 (2.1)	6.2 (1.3, 10.0)	
HSDS at start	-2.8 (0.7)	-2.6 (-5.6, -2.0)	
HSDS 1y after start GH	-2.1 (0.7)	-2.0 (-4.4, -1.0)	
HSDS 2y after start GH	-1.7 (0.7)	-1.6 (-4.2, -0.3)	
Adj-HSDS at start	-2.1 (0.9)	-2.1 (-4.7, -0.1)
Adj-HSDS 1y after start GH	-1.4 (0.8)	-1.4 (-4.0, 0.2)
Adj-HSDS 2y after start GH	-1.1 (0.8)	-1.1 (-3.6, 0.5)
Weight SDS at start	-1.8 (1.1)	-1.9 (-5.2, 1.8)
GH dose (mg/kg/wk)	0.21 (0.10)	0.21 (0.04, 0.83)	
GH max*	4.9 (3.0)	4.3 (0.47, 12.5)	
IoR first year	-0.4 (1.1)	-0.5 (-3.4, 4.1)	
IoR second year	-0.1 (1.1)	-0.1 (-3.5, 3.1)	
Adherence 0-1y	80.8 (31.1)	95.1 (0, 100)	
Adherence 1-2y	81.5 (23.0)	92.9 (0, 100)	
Adherence 0-2y	81.1 (22.2)	90.6 (7.4, 99.9)	

*Data available for n = 58

**Fig 2:**
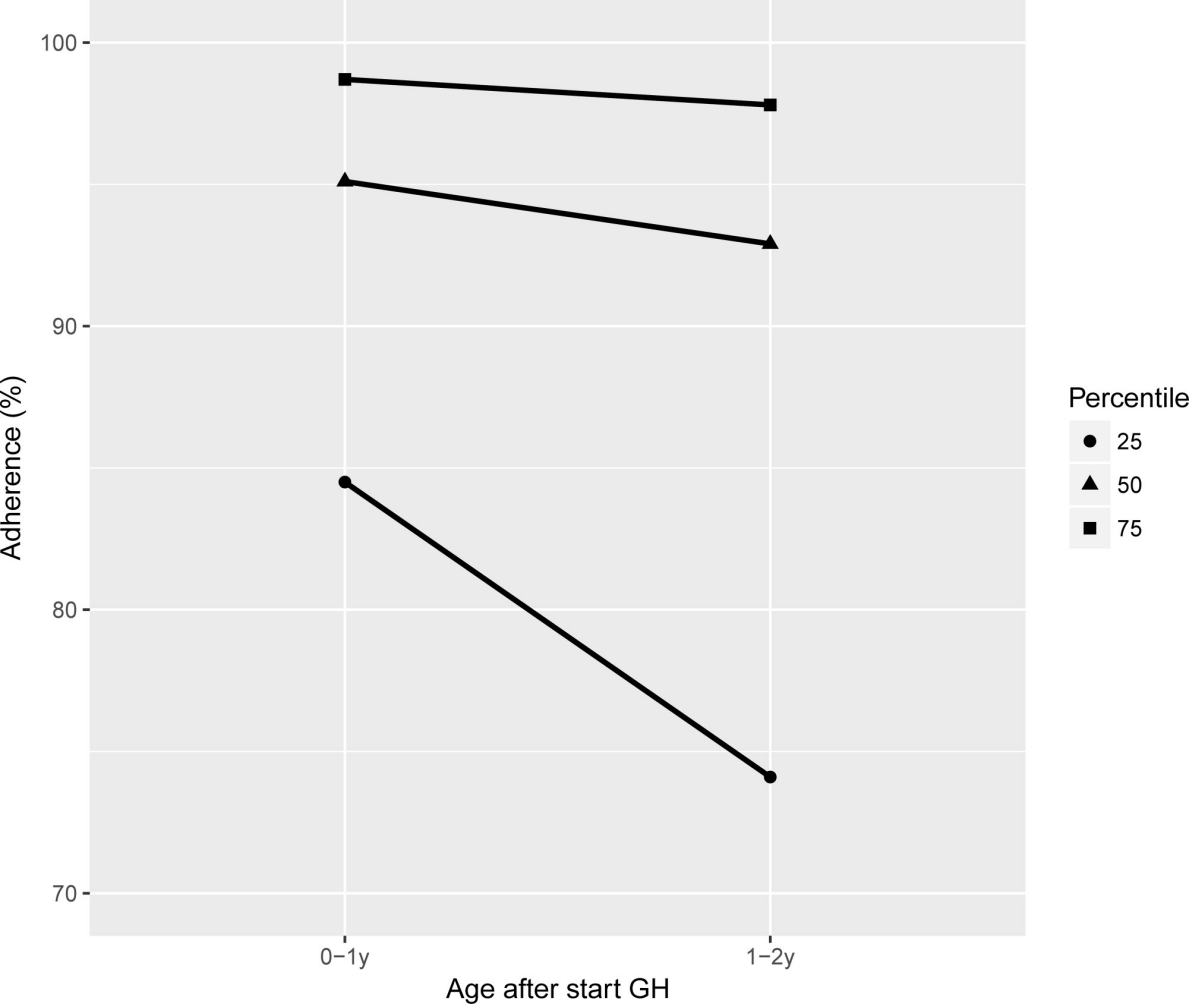
Adherence in the first two years after starting growth hormone therapy.

Fig 2 shows the median and P25-75 adherence % in the first two years of GH treatment. In the first year, median adherence was high with relatively little variation. In the second year, median adherence decreased, while the variation increased. Recursive partitioning for adherence resulted in a split at ≥98% in the first year, ≥91%, in the second year, and ≥78% in the first two years. With these splits, 32 children (34%) had a high adherence (≥98% as cut-off value) and 63 children (66%) a low adherence (<98% as cut-off value) in the first year. For the second year, these figures were 50 (53%) for a high adherence (≥91% as cut-off value) and 45 (47%) for a low adherence (<91% as cut-off value). For the first two years, these figures were 68 (72%) for a high adherence (≥78% as cut-off value) and 27 (28%) children (<78% as cut-off value).

Table 2 shows the results of the effect of adherence on ΔHSDS, the parameters of the growth curves and IoR. Ten out of eighteen associations from the final model were statistically significant. The strongest associations were found between high versus low adherence in the second year and IoR 2y, between adherence (continuous, in %) and ΔHSDS 0-2y, and between high versus low adherence in the first two years and ΔHSDS 0-2y. Mean SD) IoR at 2y was 0.19 (0.99) in the high adherence group and -0.44 (1.04) in the low adherence group. Without adjustment, mean (SD) ΔHSDS 0-2y was +1.16 (0.52) in the high adherence group and +0.88 (0.41) in the low adherence group. After adjustment for the clinical confounders, the difference in mean height between the high and low adherence group increased from +0.28 SD to +0.34 SD. The size of the effect of adherence (continuous) in the first two years on HSDS 0-2y can be calculated by multiplying the model estimate by the percentage gain of adherence. For example, a 14% lower adherence (missed 1 injection/week) in the first two years is associated with 14 x 0.00792 = 0.11 SD less height gain, and a 28% lower adherence (missed 2 injections/week) with 0.22 SDS less height gain.

**Table 2:** Results of linear regression analyses with ΔHSDS and the parameters of the growth curves as outcome and adherence as dependent variable with and without adjustment for potential clinical confounders (n = 95).

Outcome	Dependent	Model 1^a^ B (SE)	Model 2^a^ Adj. B (SE)	Model 3^a^ Adj. B (SE)	Model 4^a^ Adj. B (SE)
ΔHSDS 0-1y	Adherence 0-1y (high vs low^b^)	0.19508 (0.07277)**	0.15268 (0.06674)*	0.15281 (0.06741)*	0.156532 (0.086071)
	Adherence 0-1y (in %)	0.00114 (0.00115)	0.00101 (0.00101)	0.00111 (0.00111)	0.00193 (0.00143)
ΔHSDS 1-2y	Adherence 1-2y (high vs low^b^)	0.13908 (0.05231)**	0.15606 (0.05261)**	0.15972 (0.05250)**	0.15416 (0.05783)*
	Adherence 1-2y (in %)	0.00254 (0.00115)*	0.00282 (0.00115)*	0.00294 (0.00115)*	0.00306 (0.00113)**
ΔHSDS 0-2y	Adherence 0-2y (high vs low^b^)^	0.27744 (0.11175)*	0.29110 (0.09754)**	0.33681 (0.10161)**	0.34356 (0.12583)**
	Adherence 0-2y (in %)	0.00465 (0.00230)*	0.00500 (0.00205)*	0.00596 (0.00216)**	0.00792 (0.00253)**
A^c^	Adherence 0-2y (high vs low^b^)^	0.2199 (0.2356)	0.36290 (0.20320)	0.42619 (0.21318)*	0.38919 (0.26092)
	Adherence 0-2y (in %)	0.00406 (0.00481)	0.00887 (0.00418)*	0.01053 (0.00441)*	0.01253 (0.00518) *
k^c^	Adherence 0-2y (high vs low^b^)^	0.004739 (0.071399)	-0.02255 (0.06929)	-0.04837(0.07252)	-0.06807 (0.08505)
	Adherence 0-2y (in %)	-0.00086 (0.00145)	-0.00181 (0.00142)	-0.00256 (0.00149)	-0.00375 (0.00168)*
D^d^	Adherence 0-2y (high vs low^b^)^	0.22788 (0.07813)**	0.23054 (0.06822)**	0.24612 (0.07175)**	0.224221 (0.091026)*
	Adherence 0-2y (in %)	0.00318 (0.00163)	0.00324 (0.00146)*	0.00345 (0.00155)*	0.00408 (0.00189)*
E^d^	Adherence 0-2y (high vs low^b^)^	0.05306 (0.02061)*	0.05134 (0.01975)*	0.04698 (0.02077)*	0.03313 (0.02403)
	Adherence 0-2y (in %)	-0.00061 (0.00043)	-0.00056 (0.00042)	-0.00041 (0.00044)	-0.00030 (0.00050)
IoR^e^ 1y	Adherence 0-1y (high vs low^b^)	0.5456 (0.2442)*			
	Adherence 0-1y (in %)	-0.00299 (0.00381)			
IoR^e^ 2y	Adherence 1-2y (high vs low^b^)	0.6237 (0.2078)**			
	Adherence 1-2y (in %)	0.00882 (0.00466)			

^a^Model 1: Adherence

Model 2: Model 1 + age at start + adj-HSDS at start + birth weight SDS + gestational age (<37 weeks vs ≥37 weeks)

Model 3: Model 2 + GH dose

Model 4: Model 3 + GH max (n = 58)

^b^high ≥98%, low<98% adherence 0-1y, high ≥91%, low<91% adherence 1-2y, high ≥78%, low<78% adherence 0-2y

^Both adherence in the first and second year in the model

^c^Parameters of the monomolecular growth curve: A-5 = attained adj-HSDS after two years-5, A*(1-B)-5 = adj-HSDS at start, k = growth rate.

^d^Parameters of the second degree polynomial: D = slope (CUG), E = deceleration (deceleration after CUG)

^e^Index of Responsiveness

*p<0.05

**p<0.01

## Discussion

Our study is the first to show the effect of suboptimal adherence in a large group of patients with GHD using a reliable method to automatically assess adherence through an electronic device over the first two years of GH treatment (Easypod). The effect size of suboptimal adherence is dependent on the percentage of missed injections; if a cut-off of 78% is used, the loss of height gain was 0.34 SD, approximately 2–2.5 cm after two years of GH treatment.

It is noteworthy that average adherence in the first year was not associated with first year growth response, in contrast to the second year and both years combined. We believe that there are three explanations for this observation. The first is that average adherence in the first year is very high with little variation, which limits the power to detect statistically significant associations. Second, adherence has a skewed distribution, which may reduce the associations. Third, the effect of GH on growth is most prominent in the first year of treatment, with a relatively strong effect on growth velocity with relatively little effect of GH dose [3].

Our data show that in order to obtain a good insight into the effect of suboptimal adherence, two conditions have to be met. First, an outcome parameter has to be used that represents the natural shape of CUG. In the first years of treatment, CUG has the shape of a sharp increase of HSDS in the first year, followed by gradually decreasing height velocities until HSDS adjusted for TH-SDS is close to 0. HSDS remains stable until the onset of puberty, when the pubertal growth spurt begins. This implies that the choice for the outcome parameter is dependent on the number of years after start of GH treatment. In the first years, growth should be compared with a model for CUG, thereafter to a stable HSDS, and from pubertal onset the effect of adherence becomes impossible to analyze because of interference by the pubertal growth spurt. Our data show that over the first two years the change in HSDS gives similar or higher effects than the growth rate parameters of the two mathematical models. Although the cut-offs for adherence were based on the difference in HSDS, a similar cut-off for 0–2 y was found for the slope parameter D. We propose that this outcome measure may be superior to the various indicators of “poor response” reported in the literature [21].

The second condition that has to be met is that sufficient adjustment is made for clinical parameters that influence the growth response to GH treatment. In our study, adherence in the first two years strongly correlated with height gain 0–2 y, even unadjusted for clinical predictors. However, the effect of adherence became considerably stronger when adjustment was made to well established predictors.

While automatic recording of adherence is helpful to give insight into the frequency of suboptimal adherence and its effect on the growth response to GH treatment, for clinical care its most important benefit could be that it gives the clinician a signal to intervene and try to improve adherence. In theory, supportive accountability, including human support, motivational interviewing and communication “bandwidth” could be successful, and potentially successful approaches have been suggested [22,23,24]. However, controlled trials on the efficacy of such programs are lacking.

The limitations of this study include its non-interventional nature, which is associated with a high level of missing data, high inter-patient variability, and the absence of detailed recording of actions performed by health-care providers and carers when poor adherence and/or poor response to treatment was recorded. However, these limitations occur in all surveillance studies [25], whereas the observational nature means that it reflects normal clinical practice. Another limitation is that the cut-off values for defining a high and low adherence were constructed in the same dataset as the correlation analysis. This may have overestimated the correlations. However, similar conclusions could be drawn based on the adjusted correlations between ΔHSDS in the first, second and first two years and adherence in % (linear) and adherence in two categories (high versus low according to the cut-off values that we developed). Also, when we apply the Cutfield cut-off values [9] of high (≥86%) versus low adherence (<86%) in the first, second and first two years, similar significance levels were found with the exception of ΔHSDS in the first two years (Adj. B (SE) = 0.23156 (0.12338), p = 0.066). The cut-off value of ≥86% for a high adherence was chosen by Cutfield et al. [9], because it corresponds to no more than one missed dose a week on average. However, it is to be preferred to take the cut-off value that maximizes the correlation between adherence and height gain, because it provides more insight into the doses that are needed per several days, week or several weeks to have an optimal height gain. Further research is needed to investigate if the performance of the constructed cut-off values in our study provides similar correlations between high/low adherence and height gain in new datasets compared to our study. Another limitation of our study is that we did not include IGF-I measurements to assess GH status and predict growth response and adherence during GH therapy in our patients. IGF-I measurements were not available for the majority of patients, because ECOS was a surveillance study [26]. For this study, we only selected adherence from the easypod electronic drug-delivery device, growth outcomes and several clinical and background parameters as potential confounders.

Strengths include that it is the first study that has used a device with an eHealth platform to report adherence data directly from patients to health-care providers. A number of individual cases from ECOS have been reported [27,28,29,30]; these indicate that direct access to adherence monitoring can make the difference in a patient’s management and motivation. For example, a 14% lower adherence (missed 1 injection/week) in the first two years is associated with 14 x 0.00792 = 0.11 SD less height gain, and a 28% lower adherence (missed 2 injections/week) with 0.22 SDS less height gain.

The success of therapy depends mainly on the ability of the patients and their parents to carefully adhere to the recommended treatment regimen. Complex schedules should be avoided. This study shows that each missed injection/week in the first two years resulted in 0.11 SD less height gain, which implies that a daily routine of taking an injection, especially in the second year, is recommended. Although accurate and up-to-date adherence data can be obtained from the easypoddevice, at the moment this data is not directly available for the physician. So complete non-adherence to GH therapy is relatively easy to detect on the basis of growth failure, but suboptimal and/or intermittent adherence are more difficult to assess. Linking the adherence data from the easypod device to the physician can be helpful in the future. At the moment, regularly interviewing the patients and their parents are efficacious means of detecting the degree of adherence [31]. Different strategies can be incorporated to enhance adherence to GH therapy, i.e. providing early patient and parent education and support, medication reminder systems and longer duration of GH prescriptions [32]. A personalized approach seems to be promising, because a previous study on the ECOS data showed that several clinical parameters and background characteristics of the patients are important determinants to predict the level of adherence. Early age of self-administration, weight at start of treatment, and teenage years are associated with a lower adherence to GH treatment [33]. Patient adherence support programs that take into account these factors may improve adherence and subsequent clinical outcomes. Further studies are required to design personalized patient support programs to attain and maintain good adherence to GH treatment.

In conclusion, electronically monitoring adherence enables obtaining reliable information on adherence in a “real-life” situation, and shows a statistically significant effect on the growth response in the first two years of treatment in children with GHD. Each missed injection per week in the first two years, resulted in 0.11 SDS less height gain. The next step should be to develop interventional tools to explore the hypothesis that patient adherence support programs improve adherence and subsequent clinical outcomes.

## Supporting information

S1 Table Easypod connect observational study (ECOS) local ethical committees. (DOCX)
