# Supplementary material for: Effect of adherence to growth hormone treatment on 0–2 year catch-up growth in children with growth hormone deficiency
Source: PLoS One. 2018 Oct 24;13(10):e0206009. doi: 10.1371/journal.pone.0206009 (PMC6200242; doi:10.1371/journal.pone.0206009)
Supplement: S1 Table — (DOCX) [file pone.0206009.s001.docx]

**Easypod™ Connect Observational Study (ECOS) local ethical committees**

| **Country** | **Site Number** | **Date of Protocol Approval** | **Local Ethical Committee** |
| --- | --- | --- | --- |
| **Argentina** | 171 | 06 June 2012 | Comité independiente de ética para Ensayos en Farmacologia Clínica, |
|  | 359 | 11 February 2014 | Comité independiente de ética para Ensayos en Farmacologia Clínica |
| **Australia** | 020 | 24 December 2010 | Maters Health Services Human Research Ethics Committee |
|  | 021 | 14 February 2011 | Austin Health Human Research Ethics Committee |
|  | 022 | 1 February 2011 | ACT Health Human Research Ethics Committee. |
|  | 025 | 28 March 2011 | Monash Health Human Research Ethics Committee |
|  | 036 | 24 May 2011 | Princess Margaret Hospital for Children Ethics Committee |
| **Austria** | 018/023/026 | 21 December 2010 | Single approval for all sites by EC.  EC of the medical University Vienna and of the general hospital of the city Vienna  AKH Borschkegasse 8b / E06  A-1090 Vienna |
| **Canada** | 024 | 06 April 2011 | UBC C&W Research Ethics Board |
|  | 017 | 06 January 2011 | University of Western, Office of Research Ethics Board HSREB |
|  | 008 | 07 January 2011 | Comite d’ethique du CHU Sainte-Justine |
|  | 185 | 09 May 2012 | Service de soutien à l'éthique de la recherche en santé sur l'humain Centre hospitalier universitaire de Sherbrooke |
|  | 120 | 24 October 2011 | Health Research Ethics Board |
|  | 010 | 13 June 2011 | SickKids – Research Ethics Board |
|  | 287 | 22 March 2013 | Health Research Ethics Board – University of Manitoba |
|  | 009 | 18 November 2010 | Conjoint Health Research Ethics Board – University of Calgary |
|  | 272 | 30 April 2012 | Horizon Health Network – Research Ethics Board |
|  | 019 | 25 February 2011 | Hamilton lntegrated Research Ethics Board – McMaster University |
| **China** | 071 | 13 September 2011 | EC of the first affiliated hospital,Sun Yat-sen University |
|  | 072 | 09 August 2011 | EC of Beijing Children´s Hospital,capital medical university |
|  | 073 | 09 August 2011 | Ethics Committee of Peking union medical college hospital |
|  | 074 | 13 October 2011 | Ethics Committee of Chengdu Women & Children´s central Hospital |
|  | 103 | 12 October 2011 | Ethics Committee of Fujian province,Fuzhou Children´s Hospital |
|  | 104 | 30 November 2011 | Ethics Committee of Sun Yat-sen Memorial Hospital Sun Yat-sen University |
|  | 105 | 03 November 2011 | Ethics Committee of Children´s Hospital of Fudan University |
| **Columbia** | 361 | 20 November 2013 | Hospital San Jose Ethics Committee |
|  | 362 | 30 April 2014 | Fundación Cardio Infantil Ethics Committees |
| **Czech Republic** | 050 | 29 June 2011 | Etická komise FN Motol  V Úvalu 84, Praha 5, 150 06 |
|  | 048 | 10 June 2011 | Etická komise, Krajská zdravotní, a.s. - Masarykova nemocnice v Ústí nad Labem, o.z.  Sociální péče 3316/12A,  401 13 Ústí nad Labem |
|  | 047 | 11 July 2011 | Etická komise FN a LF UP Olomouc  I. P. Pavlova 185/6  779 00 Olomouc |
|  | 049 | 23 June 2011 | Etická komise Endokrinologického ústavu  Národní 8, 116 94 Praha 1 |
|  | 292 | 09 February 2012 | Etická komise FN Plzeň  E. Beneše 13, 305 99 Plzeň |
|  | 046 | 24 August 2011 | Etická komise FN Brno  Jihlavská 20, 625 00 Brno |
|  | 067 | 17 December 2012 | Etická komise FN Hradec Králové  Sokolská 581, 500 05 Hradec Králové |
|  | 143 | 01 February 2012 | Etická komise Fakultní nemocnice Královské Vinohrady  Šrobárova 1150/50, Praha 10, 100 34 |
| **Finland** | 331 | 21 October 2013 | Helsingin Ja Uudenmaan (local EC at site level is not applicable – Central Ethics Approval |
| **France** |  |  | CCTIRS (Comité Consultatif sur le Traitement de l'Information en Matière de Recherche dans le Domaine de la Santé). |
| **Greece** | 085 | 02 February 2011 | Local institutional review board of Athens University Medical School |
|  | 249 | 28 April 2011 | Local institutional review board of “P.&A. Kyriakou” Children’s Hospital |
|  | 252 | 28 April 2011 | Local institutional review board of “P.&A. Kyriakou” Children’s Hospital |
| **Hungary** | 144, 145, 146, 151, 152, 156, 176, 177, 204, 275 | 19 January 2012 | Central Ethics - Egészségügyi Tudományos Tanács Tudományos és Kutatásetikai Bizottsága, ETT TUKEB (Scientific and Research Ethics Committee of the Medical Research Council) |
| **Indonesia** | 371 | 22 Apriol 2013 | Health Research Ethics Committee Faculty of Medicine University of Indonesia and Cipto Mangunkusumo Hospital. |
| **Italy** | 091 | 19 October 2011 | Comitato Etico della ASL di Cagliari |
|  | 140 | 19 January 2012 | Comitato Etico dell'Università Cattolica del Sacro Cuore - Policlinico Universitario Agostino Gemelli di Roma |
|  | 092 | 14 December 2011 | Comitato Etico dell'Azienda Ospedaliera Santobono-Pausilipon di Napoli |
|  | 207 | 21 December 2011 | Comitato Etico dell'Azienda Ospedaliera Policlinico  Consorziale di Bari |
|  | 138 | 21 December 2011 | Comitato Etico della ASL LE di Lecce |
|  | 093 | 29 November 2011 | Comitato di Etica dell'IRCCS Istituto Giannina Gaslini di Genova |
|  | 180 | 10 January 2012 | Comitato Etico dell'IRCCS Centro di Riferimento  Oncologico di Basilicata di Rionero in Vulture (PZ) |
|  | 164 | 27 January 2012 | Comitato Etico dell'Azienda Ospedaliera Ospedali  Riuniti Villa Sofia-Cervello di Palermo |
|  | 345 | 11 January 2012 | Comitato Etico per le Attività Biomediche  Carlo Romano  dell'Università degli Studi Federico II di Napoli |
|  | 128 | 22 February 2012 | Comitato Etico per la Sperimentazione Clinica  dei Medicinali dell'Azienda  Ospedaliera Universitaria  Integrata di Verona |
|  | 181 | 12 March | Comitato Etico Per la Sperimentazione dell'Azienda  Ospedaliera di Padova |
|  | 183 | 24 January 2012 | Comitato Etico Unico per la Provincia di Parma |
|  | 119 | 15 December 2011 | Comitato Etico dell'IRCCS Ospedale  Casa Sollievo della Sofferenza  di S. Giovanni Rotondo (FG) |
|  | 127 | 19 December 2011 | Comitato Etico per la Sperimentazione Clinica dell IRCCS  Ospedale Pediatrico Bambino Gesù di Roma |
|  | 141 | 23 January 2012 | Comitato Etico Scientifico dell'Azienda Ospedaliera  Universitaria Policlinico Gaetano Martino di Messina |
|  | 184 | 22 March 2012 | Comitato Etico della ASL 4 di Teramo |
|  | 187 | 26 April 2012 | Comitato Etico Sperimentazione Clinica Medicinali della  AUSL 8 di Arezzo |
|  | 356 | 17 December 2013 | Comitato Etico dell'Azienda Ospedaliero-Universitaria  Ospedali Riuniti Umberto I-G.M. Lancisi-G.Salesi di Ancona |
|  | 322 | 17 December 2013 | Comitato Etico per la Sperimentazione dei Farmaci dell'  Azienda Ospedaliero-Universitaria A. Meyer  di Firenze |
|  | 327 | 19 June 2013 | Comitato Etico dell'Azienda Ospedaliera Policlinico  Consorziale di Bari |
|  | 333 | 26 June 2013 | Comitato Etico Locale per la Sperimentazione Clinica dell'  Azienda Ospedaliera L. Sacco di Milano |
|  | 325 | 08 July 2013 | Comitato Etico dell`Azienda Ospedaliera Universitaria  Policlinico Paolo Giaccone dell`Università degli Studi di  Palermo |
|  | 332 | 27 June 2013 | Comitato Etico dell'IRCCS Istituto Auxologico Italiano di Milano |
| **Kingdom of Saudi Arabia** | 368 | 16 March 2014 | Institutional Review Board  University of Dammam PO Box 2114 Dammam 31451 KSA |
|  | 351 | 07 August 2014 | Institutional Review Board  KFMC  P.O. Box 59046 Riyadh 11525  Kingdom of Saudi Arabia |
| **Korea** | 015 | 30 November 2010 | Severance Hospital Yonsei university health system Institutional Review Board |
|  | 016 | 21 October 2010 | Institutional Review Board of Ajou University Hospital |
|  | 040 | 28 July 2011 | Chonnam National University Hospital Institutional Review Board |
|  | 041 | 13 June 2011 | Institutional Review Board of Inje university Pusan Paik hospital |
|  | 042 | 28 June 2011 | Asan Medical Center Institutional Review Board |
|  | 045 | 08 August 2011 | Korea University Anam Hospital Clinical Trial Center Institutional Review Board |
|  | 043 | 10 June 2011 | Kyungpook national University Hospital Institutional Review Board |
|  | 070 | 07 November 2011 | The Catholic University of Korea Seoul ST. Mary's Hospital Institutional Review Board |
|  | 044 | 27 July 2011 | Chungbuk national university hospital Institutional Review Board |
| **Mexico** | 131, | 06 March 2012 | Comite Bioetico para la lnvestigacion Clinica S.C. |
|  | 132, 133, 134, 135, 136, 165, 166 | 14 February 2012 | Comite Bioetico para la lnvestigacion Clinica S.C. |
| **Norway** | All sites | 12 May 2014 | The Regional Committees for Medical and Health Research Ethics (REK) – Central Ethics Approval |
| **Singapore** | 375 | 17 June 2014 | SingHealth Centralised Institutional Review Board |
|  | 376 | 23 May 2014 | NHG Domain specific review board |
| **Slovakia** | All sites | 14 September | Etická komisia DFNsPCentral Ethics Approval |
| **Spain** | 190 | 4 July 2011 | Hospital General Universitario Gregorio Marañón Ethics Board |
|  | 120000985 | 24 April 2013 | Hospital Universitario De Girona Ethics Board |
| **Sweden** | 028 | 24 October 2010 | EPN - Regionala Etikprövningsnämnden i Stockholm – Central Ethics Approval |
| **Taiwan** | 343 | 26 November 2013 | Institutional Review Board of Kaohsiung Medical University Hospital |
|  | 344 | 25 December 2013 | Taipei Medical University Joint Institutional Review Board |
| **United Arab Emirates** | 349 | 01 April 2014 | Ethics and Research Committee Mafraq Hospital |
| **United Kingdom** | All sites |  | Ethics approval not required |
